# Therapeutic approaches to hypertension: A selective perspective on emerging evidence

**DOI:** 10.1515/jtim-2026-0022

**Published:** 2026-07-21

**Authors:** Lili Zhou, Toru Suzuki, Yi Zhang

**Affiliations:** Department of Cardiology, Shanghai Tenth People’s Hospital, Tongji University School of Medicine, Shanghai, China; University of Leicester, Leicester NIHR Biomedical Research Centre and BHF Centre of Research Excellence, Leicester LE1 7RH, United Kingdom

## Introduction

**Figure 1:**
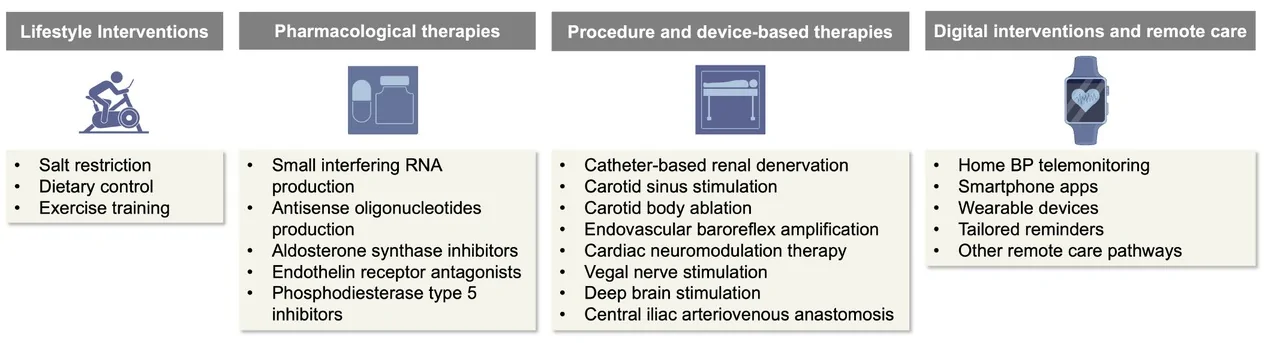
Summary of therapeutic approaches to hypertension.

Hypertension is the leading modifiable risk factor for cardiovascular morbidity and mortality worldwide^[1]^ and it drives the development of heart failure, stroke, coronary heart disease and chronic kidney disease. Contemporary management emphasizes a multimodal, precision-oriented paradigm that integrates lifestyle modification, pharmacotherapy, digital monitoring and procedural interventions. This article synthesizes developments from the past 5 years, drawing primarily from high-quality systematic reviews, meta-analyses, randomized controlled trials and contemporary hypertension guidelines. The aim is to summarize recent therapeutic approaches to hypertension (Figure 1) while providing an interpretive appraisal of their clinical implications.

## Lifestyle interventions

Nonpharmacologic strategies remain the foundation of blood pressure (BP) control. Principal measures include dietary modification and structured exercise programs. A post-hoc analysis of the DECIDE-salt trial found that progressively reducing salt intake for 2 years produced significant reductions in mean systolic BP and diastolic BP at 24 months in elderly care facilities.^[2]^ However, the study’s population, consisting exclusively of older adults residing in elderly care facilities, limits the generalizability of these findings to broader populations. Beyond individual trials, robust experimental and meta-analytic evidence has demonstrated a near-linear relationship between sodium reduction and BP lowering in both hypertensive and normotensive populations.^[3]^ On a background of salt restriction, dietary patterns such as the Dietary Approaches to Stop Hypertension (DASH) and Mediterranean diets have been associated with additional BP reductions in adults with high normal blood pressure or stage 1 hypertension,^[4]^ while evidence supporting their efficacy in individuals with moderate to severe hypertension remains limited.

In terms of physical exercise, although previous studies have clearly demonstrated that exercise training of any kind significantly reduces resting systolic and diastolic BP,^[5]^ little attention has been paid to its BP-lowering efficacy within hypertensive populations, or to the real-world barriers to implementation among patients with hypertension-related complications. These limitations are particularly relevant in hypertensive individuals with coexisting cardiac disease.

## Pharmacological therapies

Pharmacological therapy for hypertension has undergone substantial advances aimed at improving patient outcomes as well as addressing tolerability and adherence issues. These advances include the adoption of low-dose combination therapy and single-pill combinations, which provide additive effects while minimizing adverse events.^[6]^ In parallel, a number of novel pharmacologic modalities (*e.g*., small interfering RNA and antisense oligonucleotides to silence angiotensinogen, aldosterone synthase inhibitors, endothelin receptor antagonists, and phosphodiesterase type 5 inhibitors) have demonstrated BP-lowering potential in clinical trials, but most remain investigational and have not yet entered routine hypertension management.^[6]^ Among endothelin receptor antagonists, aprocitentan has been approved by the US Food and Drug Administration for the treatment of uncontrolled hypertension. These emerging therapies could broaden options for patients refractory to conventional agents, although long-term outcome evidence remains limited.

## Procedure and device-based therapies

Device-based interventions for hypertension include catheter-based renal denervation (RDN), baroreflex activation therapy, carotid body ablation, endovascular baroreflex amplification and vagal nerve stimulation.^[7]^ These methods are considered adjunctive to lifestyle and pharmacologic therapy, and reserved for patients with uncontrolled or drug-intolerant hypertension after guideline-directed treatment and multidisciplinary evaluation. RDN has become the most extensively investigated and widely adopted procedure strategy in contemporary hypertension management.

Most renal sympathetic nerves course within the periadventitial space surrounding the renal arteries and lie closer to the lumen in the distal segments. This anatomic configuration permits RDN to disrupt these fibers through endoluminal energy delivery.^[8]^ RDN reduces BP by interrupting renal sympathetic efferent and afferent signaling, thereby decreasing renal-mediated sodium and water retention, renin release, and Renin-Angiotensin-Aldosterone System activation.^[8]^ Randomized, sham-controlled trials and pooled analyses have demonstrated modest but consistent reductions in 24-hour ambulatory and office BP with RDN versus controls, with no difference observed in safety outcomes.^[9]^ However, robust evidence regarding the long-term antihypertensive effects and safety outcomes remain insufficient. The “always-on” BP-lowering effect of RDN is less susceptible to the limitations of antihypertensive medication adherence. Consequently, the 2024 ESC guideline gives RDN a Class IIb recommendation for selected patients. These include individuals who are intolerant or poorly adherent to antihypertensive drugs, or those with resistant hypertension despite optimized pharmacologic therapy, provided secondary hypertension and moderate-to-severe renal impairment have been excluded.^[10]^

A challenge across the trials of RDN lies in that roughly one-third of treated patients fail to achieve a significant response, defined as a reduction of at least 5 mmHg in ambulatory systolic BP from baseline.^[8]^ This response rate is comparable to that observed in patients undergoing percutaneous coronary intervention (PCI) for chronic coronary syndrome.^[8]^ Accordingly, the clinical value of RDN remains defensible. Non-response to RDN may reflect anatomical variability of the renal nerves, neural regeneration, incomplete lesion delivery or nerve coverage (*e.g*., lack of ablation of distal main renal arteries or branch vessels), patient phenotypes dominated by vascular stiffness (*e.g*., isolated systolic hypertension), and peri-trial medication changes or nonadherence amongst others. Developing reliable predictors to identify responders to RDN remains a key research focus.

Future research on RDN should investigate under-represented phenotypes such as isolated diastolic hypertension to clarify a wider spectrum of potential beneficiaries. Comparative investigations of different ablation energies and catheter platforms are needed to determine the most effective and durable approach, while future randomized trials should be adequately powered for hard clinical outcomes (*e.g*., major adverse cardiovascular events, mortality) rather than relying solely on BP reduction. Integrating continuous or remote BP monitoring into peri- and post-procedural management could further refine selection and enhance long-term outcomes.

## Digital interventions and remote care

Digital interventions and remote care have emerged as important adjuncts in contemporary management of hypertension by addressing several structural limitations of traditional care. Digital health solutions, such as home BP telemonitoring, smartphone apps, wearable devices, and tailored reminders, extend BP assessment beyond episodic clinic visits and facilitate more continuous patient engagement. A systematic review and meta-analysis across diverse populations indicated that digital health interventions produced greater BP reductions than standardized care.^[11]^

Compared with conventional hypertension management, digital programs incorporating automated or protocol-driven medication titration enable more timely treatment intensification and may shorten the time to blood pressure control. In addition, digital interventions can improve medication adherence, especially when monitoring data are combined with timely feedback.^[12]^ However, successful implementation depends on addressing cost, sustainability, interoperability, and scalability challenges to ensure equitable access.

## Conclusions

In summary, this perspective provides a selective interpretation of recent therapeutic advances for hypertension. First, lifestyle interventions remain foundational but need implementation frameworks to be effective at scale. Second, novel pharmacological and device-based options expand choices for refractory phenotypes but will require outcome-driven trials and better responder identification. Third, digital interventions and remote care offer transformative potential when coupled with responsive clinical workflows and attention to equity.

## References

[1] GBD 2019 Risk Factors Collaborators (2020). Global burden of 87 risk factors in 204 countries and territories, 1990-2019: a systematic analysis for the Global Burden of Disease Study 2019. Lancet.

[2] Yuan Y, Jin A, Duan P, Cao L, Wang H, Hu S (2023). Experience with 2 years’ intervention to progressively reduce salt supply to kitchens in elderly care facilities-challenges and further research: post hoc analysis of the DECIDE-Salt randomized clinical trial. BMC Med.

[3] Filippini T, Malavolti M, Whelton PK, Naska A, Orsini N, Vinceti M (2021). Blood Pressure Effects of Sodium Reduction: Dose-Response Meta-Analysis of Experimental Studies. Circulation.

[4] Filippou C, Thomopoulos C, Konstantinidis D, Siafi E, Tatakis F, Manta E (2023). DASH vs. Mediterranean diet on a salt restriction background in adults with high normal blood pressure or grade 1 hypertension: A randomized controlled trial. Clin Nutr.

[5] Edwards JJ, Deenmamode AHP, Griffiths M, Arnold O, Cooper NJ, Wiles JD (2023). Exercise training and resting blood pressure: a large-scale pairwise and network meta-analysis of randomised controlled trials. Br J Sports Med.

[6] Sayer M, Webb DJ, Dhaun N (2025). Novel pharmacological approaches to lowering blood pressure and managing hypertension. Nat Rev Cardiol.

[7] Mahfoud F, Schlaich MP, Lobo MD (2021). Device Therapy of Hypertension. Circ Res.

[8] Fisher NDL, Kirtane AJ (2025). Renal denervation for hypertension. Nat Rev Cardiol.

[9] Vukadinović D, Lauder L, Kandzari DE, Bhatt DL, Kirtane AJ, Edelman ER (2024). Effects ofCatheter-Based Renal Denervation in Hypertension: A Systematic Review and Meta-Analysis. Circulation.

[10] McEvoy JW, McCarthy CP, Bruno RM, Brouwers S, Canavan MD, Ceconi C (2024). 2024 ESC Guidelines for the management of elevated blood pressure and hypertension. Eur Heart J.

[11] Katz ME, Mszar R, Grimshaw AA, Gunderson CG, Onuma OK, Lu Y (2024). Digital Health Interventions for Hypertension Management in US Populations Experiencing Health Disparities: A Systematic Review and Meta-Analysis. JAMA Netw Open.

[12] Miao Y, Luo Y, Zhao Y, Liu M, Wang H, Wu Y (2024). Effectiveness of eHealth Interventions in Improving Medication Adherence Among Patients With Cardiovascular Disease: Systematic Review and Meta-Analysis. J Med Internet Res.

